# Optimized method for determination of 16 FDA polycyclic aromatic hydrocarbons (PAHs) in mainstream cigarette smoke by gas chromatography–mass spectrometry

**DOI:** 10.1186/s13065-018-0397-2

**Published:** 2018-03-13

**Authors:** Jana Jeffery, Maria Carradus, Karolina Songin, Michael Pettit, Karl Pettit, Christopher Wright

**Affiliations:** 1 British American Tobacco, Research and Development, Southampton, UK; 2 Marchwood Scientific Services, 371 Millbrook Rd W, Southampton, UK

**Keywords:** Polycyclic aromatic hydrocarbons (PAHs), Mainstream cigarette smoke, Gas chromatography–mass spectrometry, High resolution mass spectrometry, Low resolution mass spectrometry, Accelerated solvent extraction

## Abstract

A gas chromatography–mass spectrometry (GC–MS) method was validated for the determination of 16 polycyclic aromatic hydrocarbons (PAHs) from the FDA list of 93 harmful or potentially harmful constituents of mainstream cigarette smoke (MCS). Target analytes were extracted from total particulate matter using accelerated solvent extraction with a toluene/ethanol solvent mixture. Matrix artefacts were removed by two-step solid-phase extraction process. Three different GC–MS systems [GC–MS (single quadrupole), GC–MS/MS (triple quadrupole) and GC–HRMS (high resolution, magnetic sector)] using the same separation conditions were compared for the analysis of MCS of 3R4F Kentucky reference cigarettes generated under ISO and intense smoking regimes. The high mass resolution (m/∆m ≥ 10,000) and associated selectivity of detection by GC–HRMS provided the highest quality data for the target PAHs in MCS. Owing to the HR data acquisition mode enabling measurement of accurate mass, limits of quantification for PAHs were 5 to 15-fold lower for GC–HRMS than for GC–MS/MS and GC–MS. The presented study illustrates that the optimised sample preparation strategy followed by GC–HRMS analysis provides a fit-for-purpose and robust analytical approach allowing measurement of PAHs at (ultra)low concentrations in MCS. Furthermore, the study illustrates the importance and benefits of robust sample preparation and clean-up to compensate for limited selectivity when low-resolution MS is used.

**Electronic supplementary material:**

The online version of this article (10.1186/s13065-018-0397-2) contains supplementary material, which is available to authorized users.

## Introduction

Mainstream cigarette smoke (MCS) is an extremely complex aerosol comprising of vapour phase and particulate phase (total particulate matter, TPM) [1]. MCS contains over 6500 compounds [2], more than 100 of which are established toxicants [3].

Polycyclic aromatic hydrocarbons (PAHs) are a class of compounds containing hydrogen and carbon that comprise multiple aromatic rings. PAHs are formed during the incomplete combustion of organic material such as gas, coal, wood, tobacco and even chargrilled meat. Interestingly, PAHs do not occur naturally in tobacco plants; however, they can be introduced during tobacco curing and also deposited from vehicle exhaust during transport [4–6]. PAHs are further formed during cigarette combustion—in fact, more than 500 different PAHs have been identified in cigarette smoke at yields varying from sub-ng/cigarette to µg/cigarette [2].

In June 2009, the Family Smoking Prevention and Tobacco Control Act became law in the United States and assigned authority to the Food and Drug Administration (FDA) to regulate the manufacture, distribution and marketing of tobacco products as well as to drive requirements for testing and reporting for selected chemicals to protect public health [7]. In 2012, the FDA Tobacco Products Scientific Advisory Committee (TPSAC) established a list of 93 harmful and potentially harmful constituents (HPHCs) present in tobacco products or tobacco smoke and drafted an abbreviated list of 20 HPHCs that are required to be reported by US tobacco product manufacturers and importers [8, 9]. Although the abbreviated list contains only benzo[*a*]pyrene (B[*a*]P), the full 93 HPHC list includes 16 PAHs (naphthalene, benzo[*c*]phenanthrene, benzo[*a*]anthracene, chrysene, cyclopenta[*c,d*]pyrene, 5-methylchrysene, benzo[*b*]fluoranthene, benzo[*k*]fluoranthene, benzo[*j*]aceanthrylene, B[a]P, indeno[*1,2,3*-*cd*]pyrene, dibenzo[*ah*]anthracene, dibenzo[*a,l*]pyrene, dibenzo[*a,e*]pyrene, dibenzo[*a,i*]pyrene and dibenzo[*a,h*]pyrene) for which reporting may be required in due course.

The development of reliable methods for the quantitative measurement of PAHs in MCS at toxicologically relevant (i.e. very low) concentrations is therefore a priority. However due to the complexity of the MCS matrix and the variation of PAH concentrations, the development of such methods is challenging and very few data have been published for measurement of the full FDA suite of PAHs in MCS (most published data are for naphthalene and B[a]P only).

Several methods have been published for the quantification of PAHs in MCS using a variety of chromatographic applications, such as gas chromatography–mass spectrometry (GC–MS) [10–14], high-performance liquid chromatography (HPLC)—fluorescence detection [15–18] or tandem mass spectrometry (MS/MS) [19, 20].

There are also several GC–MS based methods for measurement of B[*a*]P in MCS adopted by laboratories in respective regions; ISO 22634 [21], which originated from CORESTA Recommended Method 58 [11], WHO TobLabNet SOP 05 [22] and Health Canada T-120 [23].

During the FDA Center for Tobacco Products (CTP) Scientific Workshop on Tobacco Product Analysis held in July 2013 [24], the suites of PAHs routinely measured by commercial testing laboratories and cigarette manufacturers were noted to differ from those in the FDA HPHCs list [8, 10, 12, 13, 19]. Furthermore, the differences in methodologies observed at the CTP meeting [24], as well as large temporal variation of of the yields of smoke constituents [25], have highlighted the need for a harmonized fit-for-purpose analytical method.

To meet the need for ultra-low quantification limits for PAHs, techniques commonly applied to trace residue analysis in regulated industries such as food and environment must be applied. These include the of stable isotope dilution and the selection of suitable solvent(s)—either a single solvent or a solvent mixture that maximises the recovery of PAHs from the MCS matrix. For example, a solvent mixture combining polar and non-polar solvents was reported to increase PAH recoveries from soot, sediment and Standard Reference Material (SRM) diesel particulate matter [26, 27]. Additionally, chromatographic selectivity can be optimised by using the most appropriate GC stationary phase (e.g., DB-EUPAH, which was developed specifically for the separation of PAHs) [28]. In some cases, low-resolution mass spectrometers may not achieve the required quantification limits and more sensitive detection may be required. Alternatively, thorough and highly selective sample preparation and clean up may remove enough chemical background to enable the use of low-resolution MS if high-resolution MS is not available.

The aim of the present study was to evaluate an analytical method and to compare three GC–MS systems for the measurement of the 16 PAHs of the FDA HPHC list (GC–MS, GC–MS/MS and GC–HRMS). To our knowledge, this is the first study of measurement of all FDA specified PAHs in MCS for which the majority of data exceed the limit of quantification.

## Experimental

### Materials

Glass fibre filter pads (92-mm; Cambridge filter pads, CFPs) were purchased from Borgwaldt KC (Hamburg, Germany). University of Kentucky 3R4F reference cigarettes were obtained from the Center for Tobacco Reference Products (University of Kentucky, USA), see Table 1 for 3R4F main characteristics [29]. Base-modified silica cartridges 70 ml/10 g were sourced from Biotage (Uppsala, Sweden).

**Table 1:** 3R4F Kentucky reference cigarette main characteristics

Parameter	Mean value (mg/cigarette)
Weight	1060
TPM [30]	11.0
Nicotine [31]	0.73
NFDPM [30]	10.27
CO [32]	12.0
Puff count [33]	9.0

*TPM* total particulate matter, *NFPDM* nicotine-free dry particulate matter (TPM with nicotine and water subtracted; ‘tar’)

### Chemicals

As mentioned in the Introduction, there are 16 PAH substances are on FDA HPHC list (Additional file 1: Figure S1). PAH calibration solutions were obtained from Wellington Laboratories (Guelph, Canada) and contained a mixture of native and deuterium (D)-labelled PAHs, and internal standards (Additional file 1: Table S1). The native standards were supplied at concentrations of 2, 10, 40, 200 and 800 ng/ml (product codes PAH-A-CS1, PAH-A-CS2, PAH-A-CS3, PAH-A-CS4 and PAH-A-CS5, respectively); each solution contained the mass labelled analogues each at 100 ng/ml. The standard mixes were supplied in toluene/isooctane containing toluene at 2, 2.1, 2.4, 4 and 10%, respectively.

Mixed solutions containing only the D-labelled PAHs at 2000 ng/ml (product code PAH-CVS-A) or internal standards at 2000 ng/ml (PAH-ISS-A) were also obtained from Wellington Laboratories. The PAH-CVS-A standard was diluted in toluene:isooctane (2:98, v/v) to obtain standards of lower concentration for GC–HRMS calibration. The D-labelled internal standards (from PAH-ISS-A) were prepared at 100 ng/ml in isooctane:toluene (75:25, v/v).

A mixed solution of 16 ^13^C-labelled PAHs at 5 µg/ml; 99% purity in nonane (US EPA 16 PAH; product code ES-4087) was obtained from Cambridge Isotope Laboratories (Tewksbury MA, USA; Additional file 1: Table S2). The following individual standards also from Cambridge Isotope Laboratories were used as well: dibenzo[*a,e*]pyrene-^13^C_6_ (chemical purity 96.3%), 100 µg/ml in *n*-nonane:distilled toluene (80:20) (product code CLM-3835-1.2); dibenzo[*a,i*]pyrene-^13^C_12_ 50 µg/ml in nonane (chemical purity 99.2%, product code CLLM-3774-A-T-S); and benzo[*e*]pyrene-9,10,11,12-^13^C_4_ 100 µg/ml in nonane (chemical purity 99%, product code CLM-6170-S). In addition, a benz[*j*]aceanthrylene-^13^C_2_,d_2_ and benz[*e*]aceanthrylene-^13^C_2_,d_2_ standard mix (product code B197912), and a mixture of benz[*j*]aceanthrylene to benz[*e*]aceanthrylene in the ratio of 70:30 (product code B197910), both with chemical purity of all compounds of 98% were obtained from Toronto Research Chemicals (North York, Canada).

All solvents (ethanol, toluene, cyclohexane) were analytical grade and purchased from Rathburn Chemicals (Walkerburn, UK). Silica was obtained from MP Biomedicals (Loughborough, UK). All other reagents including concentrated formic acid were analytical grade and purchased from Sigma Aldrich (Gillingham, UK).

### Samples

The test cigarettes 3R4F and CFPs were conditioned per ISO 3402 (22 ± 1 °C and 60 ± 3% relative humidity for a minimum of 48 h but not exceeding 10 days) to ensure their consistency [30, 34]. Total Particulate Matter (TPM) was collected on 92 mm Cambridge Filter Pads by smoking 20 or 10 cigarettes under ISO [35] or Health Canada Intense T-115 (HCI, vents fully blocked) smoking regimes [36], respectively, using a rotary smoking machine RM200A (Borgwaldt KC, Hamburg, Germany). CFPs were stored in 60 ml amber glass containers in the freezer set at − 20 °C until extraction and analysis.

### Sample extraction and clean-up

Before extraction, each CFP was fortified with 100 ng of D-labelled and ^13^C-labelled PAH internal standards in cyclohexane and left to equilibrate for 24 h in the refrigerator set at 4 °C. Sample extraction was performed by Accelerated Solvent Extraction (ASE) using a Buchi 916 instrument with a 40-ml cell (Buchi, Oldham, UK). A single cycle of ASE was used to extract the CFP in 40 ml of solvent (ethanol/toluene 1:9, v/v) at 100 °C with a hold time of 5 min.

For sample clean-up, 4 ml of the CFP extract was added to 20 ml of concentrated formic acid. The mixture was shaken for 2 min on a laboratory shaker set at 300 rpm, and then centrifuged for 5 min at 1500 rpm for phase partitioning. The upper organic layer was removed and retained, and 25 ml of toluene was added to the aqueous layer, which was then shaken and centrifuged as above. The upper layer was again removed and added to the first organic layer. The combined organic extract was added to 25 ml of concentrated formic acid and shaken for 2 min at 300 rpm; 20 ml of water was then added, and the extract was shaken for a further 2 min. Samples were then centrifuged for 5 min at 1500 rpm to allow phase partitioning. The upper organic layer was removed and filtered through sodium sulphate and concentrated to 5 ml using a rotary evaporator set at 40 °C.

**Fig. 1:**
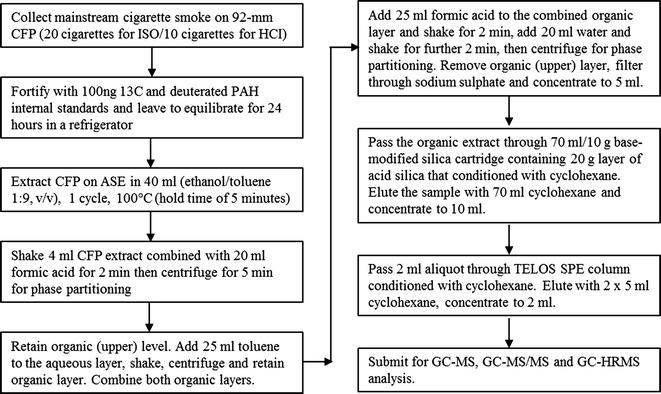
Flow chart of analytical procedure

The organic extract was first passed through a 70 ml/10 g base-modified silica cartridge containing 20 g layer of acid silica [prepared by mixing 100 g of silica (MP Biomedicals, Loughborough, UK) with 40 g of formic acid]. The column was pre-washed with 70 ml of cyclohexane, the sample was loaded and then eluted with 70 ml of cyclohexane. The eluate was collected and concentrated to 10 ml. Aliquots of this sample (2 ml) were passed through a TELOS Solid-Phase Extraction (SPE) column 1.5 g/6 ml (Part No. 550-015G-006T, Kinesis, St Neots, UK) conditioned with cyclohexane. The column was eluted with 2 × 5 ml of cyclohexane, and the eluate was concentrated to 2 ml final volume. To ensure consistency of the sample and minimise any variations, the extract was then divided into three aliquots for the analysis by gas chromatography–mass spectrometry (GC–MS). GC–MS systems with three different mass analysers were compared: low resolution with a single quadrupole (GC–MS), low resolution with triple quadrupole (GC–MS/MS) and high resolution with double-focussing magnetic sector (GC–HRMS). A schematic flow chart of the analytical procedure is summarised in Fig. 1.

### GC separation conditions

The same separation conditions were used for all three types of MS analysis^1^ (Table 2). For GC–MS/MS only, QQQ collision cell, EPC helium quench gas flow was 2.25 ml/min and N_2_ collision gas flow was 1.5 ml/min.

**Table 2:** GC conditions used for analysis of PAHs in mainstream smoke

GC separation conditions	
Injection	Multimode (PTV) injection, splitless mode
Injection volume	2 µl
Carrier gas	Helium; 1 ml/min (50 min), then 2 ml/min (until the end of the analytical run)
Column	Agilent DB-EUPAH (60 m × 250 mm × 0.25 µm)
Oven temperature programme	50 °C (0.8 min), ramp 45 °C/min to 200 °C, ramp 2.5 °C/min to 225 °C, ramp 5 °C/min to 266 °C, ramp 14 °C/min to 300 °C, ramp 10 °C/min up to 320 °C (48 min). Total run time 74.762 min

### Mass spectrometry

The single-quadrupole mass analyser used for GC–MS was an Agilent Technologies 6890N GC system coupled to an Agilent 5973N Quadrupole Mass Spectrometer with Agilent Mass Hunter Version E.02.1431 (Agilent Technologies, Wokingham, UK). The triple-quadrupole mass analyser used for GC–MS/MS was an Agilent 7890N with Mass Hunter software version B05.02. The magnetic sector high-resolution mass spectrometer used for GC–HRMS was an Agilent 6890N GC system coupled to a Waters AutoSpec P716 HRMS with MassLynx software version 4.1 SCN815 (Waters, Elstree, UK). The MS data acquisition parameters for GC–MS, GC–MS/MS and GC–HRMS are presented in Additional file 1: Tables S3–S7.

### Data analysis

Data analysis was conducted using the above-mentioned software.

### Quality assurance

Unfortified CFPs were extracted to provide method blank samples. For regular monitoring of analytical method performance, unsmoked/blank CFPs were fortified with 40 ng of native standards, 100 ng of internal standards and extracted following the analytical procedure (Additional file 1: Table S8). Recoveries of native PAHs in quality control samples were calculated by division of the mass of PAHs quantified per CFP by the fortification mass. Values were multiplied by 100 to obtain the percentage recovery. Internal standards recovery was assessed for each analytical sequence to monitor the method performance.

The limit of quantitation (LOQ) was established as the lowest concentration of an analyte in a sample that can be determined with acceptable precision and accuracy under the stated conditions of test [37]. The LOQs were determined for each MS system from the respective S/N ratio of each analyte in 3R4F mainstream smoke extract to represent analytical conditions.

## Results and discussion

The complexity of mainstream smoke can result in a multitude of co-extracted matrix components that may significantly compromise the analysis. As mentioned in the introduction, thorough optimisation of several key aspects of an analytical method is critical to achieve the required selectivity and sensitivity.

### Solvent selection

Initially, methanol and cyclohexane were assessed as the most frequently referenced solvents for extraction of PAHs. Visual inspection of the CFP after extraction indicated that a more polar solvent such as methanol might extract TPM more efficiently from the CFP (the pad appeared visually clean after extraction) compared with the non-polar cyclohexane (TPM residues remained visible on the pad). However, several papers reported advantages of using a mixture of polar and non-polar solvents for gaining higher recoveries of PAHs from complex matrices such as soot and diesel particulate matter [26, 27]. For example, Masala et al. [27] reported 2–17× higher concentrations of PAHs found in diesel particular matter when a solvent system of toluene/ethanol (9:1, v/v) coupled to ASE was used compared to toluene [27]. Therefore, toluene/ethanol (9:1, v/v) was selected.

### Signal-to-noise ratio

**Fig. 2:**
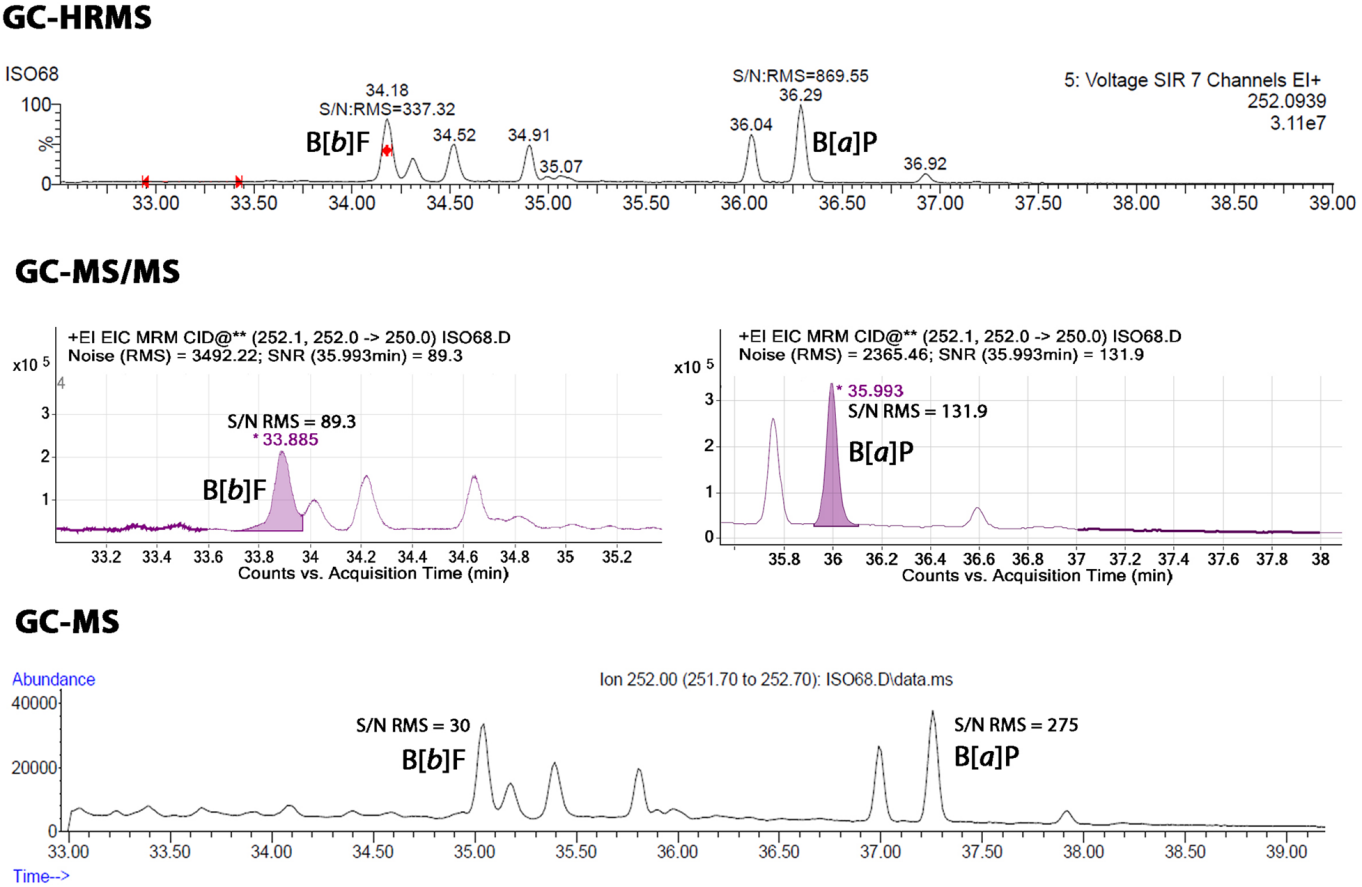
Benzo[*b*]fluoranthene and B[*a*]P separation and sensitivity (S/N) on tested GC/MS systems in 3R4F ISO MCS

The signal-to-noise ratios (S/N) were calculated using the respective instrument software. The baseline segments for estimation of noise were auto-selected and noise was calculated as the root-mean-square (RMS) of the baseline over the selected time window. A higher S/N ratio was observed for GC–HRMS and GC–MS/MS than for GC–MS for the TPM extracts. Examples of the S/N ratios observed for early, mid and late eluting compounds in 3R4F MSC are shown in Table 3. As expected, GC–HRMS gave the highest S/N ratios for the majority of PAHs, indicating the highest sensitivity and therefore the ability to measure all target analytes at required low levels. For example, for B[*a*]P, the S/N achieved by GC–HRMS was 3–7 times higher than those achieved by either GC–MS or GC–MS/MS, respectively. S/N for late eluting 6-ring dibenzopyrenes was 1–3 times higher from GC–HRMS compared to GC–MS and GC–MS/MS. An example of chromatographic separation and S/N for benzo[*b*]fluoranthene and B[*a*]P on all three GC/MS systems is shown at Fig. 2. All three instruments had the same GC separation conditions and were equipped with a DB-EUPAH capillary column specifically designed for optimal separation of PAHs.

**Table 3:** Signal/noise ratios observed for early, mid and late eluting compounds in 3R4F ISO mainstream smoke

Ion (*m/z*)	PAH	GC–HRMS		GC–MS/MS		GC–MS	
		Retention time (min)	S/N	Retention time (min)	S/N	Retention time (min)	S/N
128	Naphthalene	7.5	5332	7.3	228	7.8	1107
136	Naphthalene-d_8_	7.5	315	7.3	627	7.7	4
134	^13^C_6_-Naphthalene	7.5	1927	7.4	54	7.8	4
252	Benzo[*a*]pyrene	36.3	870	35.9	132	37.3	275
264	d_12_-Benzo[*a*]pyrene	36.1	1976	35.9	518	37.1	143
256	^13^C_4_-Benzo[*a*]pyrene	36.3	1250	35.9	759	37.3	72
278	Dibenzo[*a,h*]anthracene	43.3	362	42.6	114	48	39
302	Dibenzo[*a,l*]pyrene	61.1	56	59.2	21	63	58
302	Dibenzo[*a,e*]pyrene	66.6	114	64.5	43	68.8	98
302	Dibenzo[*a,i*]pyrene	70.3	64	67.9	19	72.6	44
302	Dibenzo[*a,h*]pyrene	72.5	43	69.9	14	74.9	20
314	^13^C_12_-Dibenzo[*a,i*]pyrene	70.3	191	67.9	217	72.6	106

### Limit of quantification (LOQ)

For each MS system, the LOQ was calculated in ng/CFP from the analyte concentration and respective S/N ratio. The LOQ per cigarette was then estimated using the number of cigarettes smoked (Table 4). As can be seen on Table 4, LOQs for PAHs obtained by GC–HRMS were 5 to 15-fold lower compared to lower resolution mass analysers, this is due to high resolution power and high mass accuracy of GC–HRMS enabling to distinguish two peaks of slightly different mass-to-charge ratios. This increases selectivity and sensitivity in complex matrices (especially when trace analysis is required), which was a significant requirement for this study.

**Table 4:** Comparison of LOQs for 16 PAHs achieved by GC–HRMS, GC–MS/MS and GC–MS

Analytes	GC–HRMS		GC–MS/MS		GC–MS	
	LOQ, (ng/CFP^a^)	Estimated LOQ, (ng/cig)	LOQ, (ng/CFP^a^)	Estimated LOQ, (ng/cig)	LOQ, (ng/CFP^a^)	Estimated LOQ, (ng/cig)
Naphthalene	0.51	0.026	1178.71	58.94	108.17	5.41
Benzo[*c*]phenanthrene	0.04	0.002	ND	ND	66.80	3.34
Benzo[*a*]anthracene	0.03	0.002	38.57	1.93	38.11	1.91
Chrysene	0.04	0.002	50.13	2.51	49.61	2.48
Cyclopenta[*c,d*]pyrene	0.02	0.001	48.84	2.44	60.04	3.00
5-Methylchrysene	0.04	0.002	ND	ND	2.48	0.12
Benzo[*b*]fluoranthene	0.04	0.002	11.44	0.57	5.08	0.25
Benzo[*k*]fluoranthene	0.05	0.003	12.41	0.62	5.07	0.25
Benzo[*j*]aceanthrylene	0.09	0.005	ND	ND	ND	ND
Benzo[a]pyrene	0.04	0.002	5.01	0.25	3.03	0.15
Indeno[*1,2,3*-*c,d*]pyrene	0.02	0.001	5.46	0.27	1.54	0.08
Dibenzo[*a,h*]anthracene	0.07	0.004	0.83	0.04	1.48	0.07
Dibenzo[*a,l*]pyrene	0.05	0.003	ND	ND	ND	ND
Dibenzo[*a,e*]pyrene	0.04	0.002	0.80	0.04	0.28	0.01
Dibenzo[*a,i*]pyrene	0.06	0.003	1.33	0.07	ND	ND
Dibenzo[*a,h*]pyrene	0.07	0.004	2.99	0.15	ND	ND

*ND* analyte not detected in the sample

^a^20 cigarettes per CFP were smoked under ISO smoking regime

The LOQs for GC–MS and GC–MS/MS were of a similar order of magnitude compared to GC/MS published data [13]. Ding et al. reported limits of detection (LODs) between 0.01 and 0.1 ng/cigarette from blank CFP (i.e. no smoke matrix) fortified with PAHs using HPLC–MS/MS [19].

### Quantification of PAHs by GC–HRMS, GC–MS/MS and GC–MS

The PAH levels in the TPM of 3R4F cigarettes smoked under both ISO and HCI conditions were quantified by the three types of GC–MS using ^13^C-labelled standards for calibration. The recovery of the internal standards was also calculated by dividing the peak area of the internal standard in each replicate by the average peak area obtained for the calibration standard. As mentioned in “Experimental” section, the same extracts were analysed on all three GC–MS systems. The recoveries of internal standards as measured by the different methods are compared in Additional file 1: Tables S9 and S10. Although in general, the apparent recoveries were comparable between the three GC–MS systems, some internal standards (e.g. naphthalene, benzo[*j*]aceanthrylene, dibenzo[*ah*]anthracene) had consistently lower recoveries for both smoking regimes in both low resolution systems. The recoveries were the most stable and consistent in GC–HRMS, therefore GC–HRMS accuracy and precision data were used in the text below as examples illustrating method performance. For 3R4F ISO mainstream smoke, internal standard recoveries ranged from 66% (benzo[*j*]aceanthrylene) to 86% (dibenzo[*a,i*]pyrene) and the repeatability from 3% (benzo[*a*]anthracene, B[*a*]P) to 13% (dibenzo[*a,i*]pyrene). Similar results were obtained in the case of 3R4F HCI mainstream smoke with internal standard recoveries 66% (dibenzo[*ah*]anthracene) to 92% (benzo[*b*]fluoranthene and benzo[*j*]fluoranthene) and repeatability from 4% (naphthalene) to 12% (benzo[*b*]fluoranthene).

**Fig. 3:**
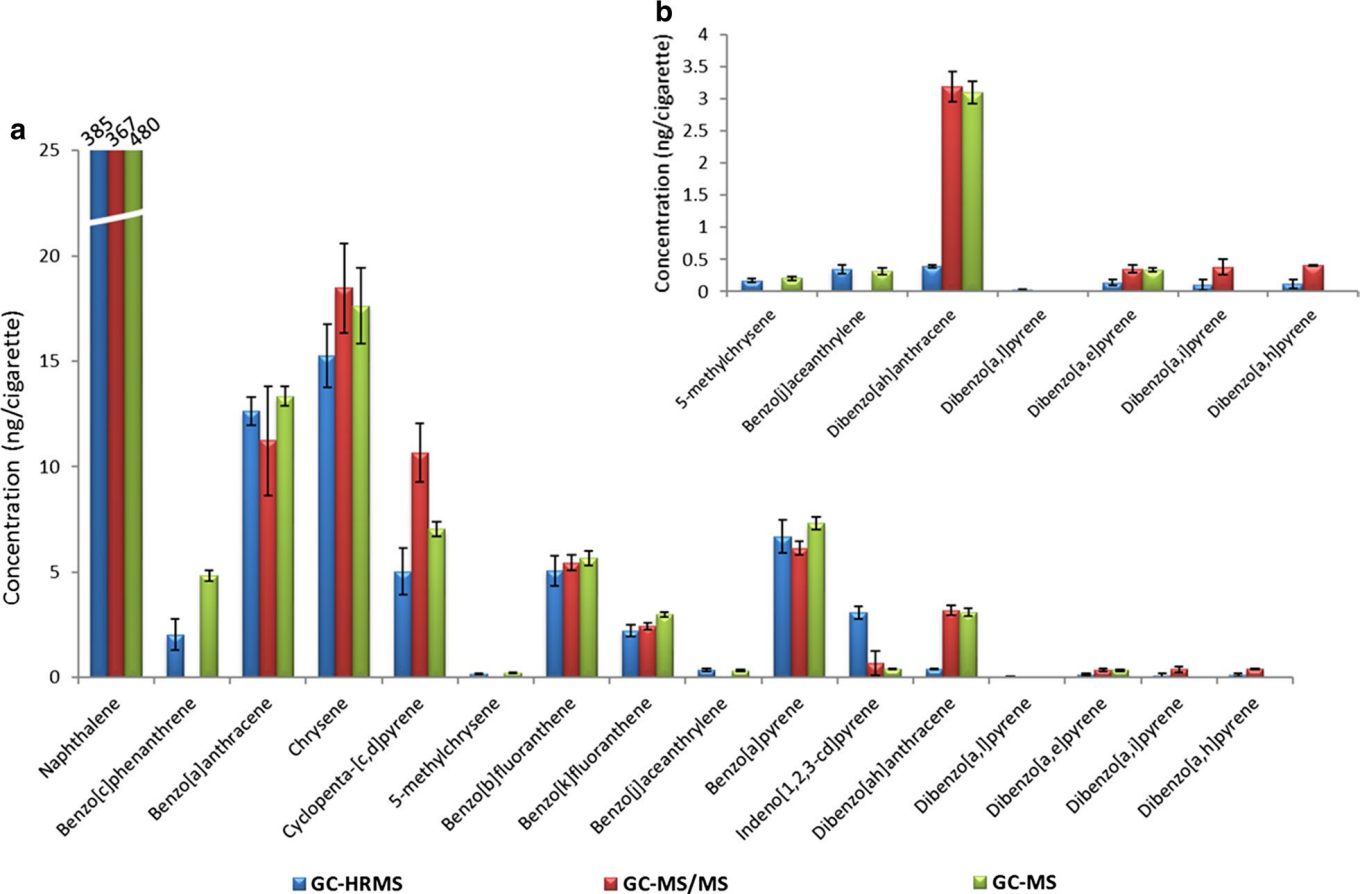
PAHs in 3R4F ISO MCS (**a**). Zoom view PAHs at (ultra)low levels (**b**)

For the ISO TPM extracts, all 16 analytes were quantified by GC–HRMS (Table 5). In contrast, four PAHs were below the LOQ for GC–MS/MS analysis (benzo[*c*]phenanthrene, 5-methylchrysene, benzo[*j*]aceanthrylene and dibenzo[*a,l*]pyrene), and three were not detected by GC–MS (dibenzo[*a,l*]pyrene, dibenzo[*a,i*]pyrene and dibenzo[*a,h*]pyrene). The mean yields (6 replicates) of detected analytes were comparable between the three GC–MS techniques and were also comparable to the limited published data that are available (Table 5) [10, 13, 38]. For example, Roemer et al. [38] reported the concentrations of PAHs in the smoke of 2R4F and 3R4F cigarettes, but with the exception of dibenzo[*a,e*]pyrene, the dibenzopyrenes were all below the limit of quantification. Dibenzo[*a,h*]anthracene, dibenzo[*a,l*]pyrene, dibenzo[*a,e*]pyrene, dibenzo[*a,i*]pyrene and dibenzo[*a,h*]pyrene yields were lower for GC–HRMS than for GC–MS/MS or GC–MS. This might be due to the higher selectivity of the HR instrument and associated removal of matrix contributions to the signal for some analytes. The repeatability of six replicates, expressed as the relative standard deviation (RSD,  %) was expected to be the poorest for PAHs present at sub-ng levels (dibenzopyrenes) and remaining analytes had RSDs largely less than 20%. Figure 3 shows a graphical comparison of PAHs measured in 3R4F ISO mainstream smoke by all three GC/MS systems (presented are mean values, n = 6 replicates).

**Table 5:** PAH levels in 3R4F ISO MCS obtained by three GC/MS systems using ^13^C-labelled internal standards

PAH	GC–HRMS		GC–MS/MS		GC–MS		IS recovery^a^		Published values, (ng/cig) [10]	Published values, (ng/cig) [13]	Published values, (ng/cig) [37]
	Mean, (ng/cig)^b^	RSD, (%)	Mean, (ng/cig)^b^	RSD, (%)	Mean, (ng/cig)^b^	RSD, (%)	Mean, (%)	RSD, (%)			
Naphthalene	385.3	7	367.3	6	479.6	22	73	7	287 ± 36	360.8	NR
Benzo[*c*]phenanthrene	2.0	35	< 0.5	NA	4.8	5	–^c^	–	NR	NR	NR
Benzo[*a*]anthracene	12.6	5	11.2	23	13.3	3	81	3	13 ± 1.4	14.1	11.8
Chrysene	15.3	10	18.5	12	17.6	10	59	4	17 ± 1.8	16.2	NR
Cyclopenta-[*c,d*]pyrene	5.0	22	10.7	13	7.0	5	–	–	NR	NR	NR
5-Methylchrysene	0.2	14	< 1.5	NA	0.2	13	–	–	NR	NR	< 0.4
Benzo[*b*]fluoranthene	5.1	14	5.5	7	5.7	6	79	4	8.3 ± 1.0	5.4	5.09
Benzo[*k*]fluoranthene	2.2	13	2.4	6	2.9	4	83	3	1.6 ± 0.3	2.2	2.02
Benzo[*j*]aceanthrylene	0.4	20	< 2.5	NA	0.3	15	66	8	NR	NR	NR
Benzo[*a*]pyrene	6.7	12	6.2	5	7.3	4	75	3	7.0 ± 0.8	6.6	6.73
Indeno[*1,2,3*-*cd*]pyrene	3.1	9	0.7	85	0.4	6	78	5	NR	3.8	2.87
Dibenzo[*ah*]anthracene	0.4	6	3.2	7	3.1	6	80	4	NR	NR	< 0.97
Dibenzo[*a,l*]pyrene	0.03	34	< 7.5	NA	< 0.2	NA	–	–	NR	NR	< 0.19
Dibenzo[*a,e*]pyrene	0.1	32	0.4	16	0.3	8	82	10	NR	NR	0.173
Dibenzo[*a,i*]pyrene	0.1	79	0.4	32	< 0.2	NA	86	13	NR	NR	< 0.22
Dibenzo[*a,h*]pyrene	0.1	59	0.4	2	< 0.2	NA	–	–	NR	NR	< 0.23

*IS* internal standard, *NA* not applicable, *NR* not reported, *RSD* relative standard deviation

^a^Recovery calculated from GC–HRMS data

^b^n = 6 replicates

^c^^13^C mass labelled internal standards were not available

Similar results were obtained for the 3R4F HCI extracts; all analytes were quantifiable by GC–HRMS (Table 6) [38], whereas three were below the LOQ by GC–MS/MS (5-methylchrysene, benzo[*j*]aceanthrylene and dibenzo[*a,l*]pyrene), and three were not detected by GC–MS at all (dibenzo[*a,l*]pyrene, dibenzo[a,i]pyrene and dibenzo[a,h]pyrene). The PAH yields were comparable among the three techniques and with published data (Table 6) [38], although the information on HCI yields is very sparse.

**Table 6:** PAH levels in 3R4F HCI MCS obtained by three GC/MS systems using ^13^C-labelled internal standards

PAH compound	GC–HRMS		GC–MS/MS GC–MS				IS recovery^a^		Published data, (ng/cig) [38]
	Mean, (ng/cig)^b^	RSD, (%)	Mean, (ng/cig)^b^	RSD, (%)	Mean, (ng/cig)^b^	RSD, (%)	Mean, (%)	RSD, (%)	
Naphthalene	1249	13	1197	10	1564	9	73	4	NR
Benzo[*c*]phenanthrene	4.4	40	7.6	45	8.5	14	–^c^	–	NR
Benzo[*a*]anthracene	30.6	11	33.0	6	29.8	5	87	10	29.80
Chrysene	35.3	12	38.3	9	35.9	3	75	11	NR
Cyclopenta-[*c,d*]pyrene	9.7	17	19.1	16	17.5	4	–	–	NR
5-Methylchrysene	0.4	10	< 1.5	NA	0.4	7	–	–	< 0.1
Benzo[*b*]fluoranthene	12.1	14	13.1	7	12.6	2	92	12	13.20
Benzo[*k*]fluoranthene	5.1	9	5.7	7	5.5	7	92	11	5.38
Benzo[*j*]aceanthrylene	0.7	28	< 2.5	NA	0.5	39	82	1	NR
Benzo[*a*]pyrene	15.2	11	13.9	7	16.2	9	85	6	16.20
Indeno[*1,2,3*-*cd*]pyrene	6.8	10	1.2	13	0.8	9	70	6	7.37
Dibenzo[*ah*]anthracene	0.9	9	7.1	10	7.0	2	66	5	< 2.4
Dibenzo[*a,l*]pyrene	0.1	19	< 7.5	NA	< 25	–	–	–	< 0.475
Dibenzo[*a,e*]pyrene	0.5	30	0.8	7	0.7	5	70	6	0.86
Dibenzo[*a,i*]pyrene	0.4	46	0.8	30	< 75	–	75	6	< 0.55
Dibenzo[*a,h*]pyrene	0.3	27	1.1	29	< 75	–	–	–	< 0.575

*IS* internal standard, *NA* not applicable, *NR* not reported, *RSD* relative standard deviation

^a^Recovery calculated from GC–HRMS data

^b^n = 6 replicates

^c^^13^C mass labelled internal standards were not available

Because of its high mass resolution (M/∆M ≥ 10,000), accurate mass (typically < 5 ppm accuracy) and associated high selectivity of detection, GC–HRMS provided the highest quality data, which were reflected in the ability of GC–HRMS to quantitatively measure all 16 PAHs in complex mainstream smoke compared to both low resolution systems. The comparative limitations of GC–MS/MS and GC-LRMS were illustrated by the case of dibenzopyrene isomers, which are present at low levels and may contribute to overall toxicity but are commonly reported as non-detect results.

The availability of quantitative data is especially relevant for toxicologically significant PAHs such as dibenzo[*j*]aceanthrylene and dibenzopyrene isomers (dibenzo[*a,l*]pyrene, dibenzo[*a,e*]pyrene, dibenzo[*a,i*]pyrene and dibenzo[*a,h*]pyrene).

### Quantification using deuterated (D) and ^13^C calibration

Stable isotope dilution is a robust technique of measurement by ratio [39]. Deuterium-labelled analogues are typically less expensive and more commercially available with shorter lead times compared to ^13^C-labelled analogues. However, ^13^C-labelled analogues are not affected by deuterium–proton exchange and have similar mass spectra to the native substance (deuterated analogues can undergo different mass losses if a deuterated moiety fragments) [40, 41]. Although in theory a single labelled analogue per homologue group is acceptable, in practice a labelled analogue per target substance accounts more fully for any matrix artefacts.

D- and ^13^C-labelled internal standards calibration was compared for quantification of PAH yields by GC–HRMS. Both quantification methods produced comparable masses of PAH compounds in 3R4F mainstream cigarette smoke generated under ISO and HCI conditions (Table 7) indicating consistency between both methods of calibration. RSD values for both D- and ^13^C calibrations were broadly comparable between both ISO and HCI sample sets. Interestingly, in ISO extracts, RSDs for some analytes including dibenzopyrenes were higher when D-labelled calibration was used compared to ^13^C. In HCI extracts, the opposite trend was observed. RSDs of < 20% was observed for all PAH compounds quantified using D-labelled analogues as the internal standards apart of dibenzo[*a,e*]pyrene (22%). For ^13^C-HCI quantitation, the RSD was < 10% for all analytes except dibenzo[*a,l*]pyrene (RSD, 16%). The RSD was < 15% for 11 and 9 of the 16 analytes using D- and ^13^C-labelled calibration, respectively. Calibration was observed to be generally consistent for most compounds using either set of mass-labelled internal standards.

**Table 7:** PAH levels in 3R4F MCS quantified by GC–HRMS using D- or ^13^C internal standards

PAH compound	ISO smoking regime						HCI smoking regime					
	D-labelled IS		^13^C-labelled IS		IS recovery^c^		D-labelled IS		^13^C-labelled IS		IS recovery^c^	
	Mean, (ng/cig)^a^	RSD, (%)	Mean, (ng/cig)^b^	RSD, (%)	Mean, (%)^b^	RSD, (%)	Mean, (ng/cig)^c^	RSD, (%)	Mean, (ng/cig)^b^	RSD, (%)	Mean, (%)^b^	RSD, (%)
Naphthalene	369.8	3	372.2	4	73.0	7	1326	7	1381	3	72.8	4
Benzo[*c*]phenanthrene	1.6	16	3.1	9	NC	NC	3.9	17	8.2	4	NC	NC
Benzo[*a*]anthracene	12.4	5	12.5	2	81.0	3	28.0	3	29.3	2	86.8	10
Chrysene	14.1	8	14.6	13	59.0	4	31.0	8	37.0	4	74.6	11
Cyclopenta-[*c,d*]pyrene	4.0	12	4.3	19	NC	NC	7.6	5	10.0	6	NC	NC
5-Methylchrysene	0.2	9	0.2	11	NC	NC	0.4	9	0.4	8	NC	NC
Benzo[*b*]fluoranthene	4.4	10	5	8	79.3	4	10.4	13	13.9	2	92.4	12
Benzo[*k*]fluoranthene	2.0	12	2.1	4	82.7	3	4.7	9	5.4	2	91.5	11
Benzo[*j*]aceanthrylene	0.3	15	0.4	17	66.3	8	0.6	12	0.8	7	81.7	5
Benzo[*a*]pyrene	6.0	11	6.4	2	75.0	3	13.6	14	15.9	3	84.6	6
Indeno[*1,2,3*-*cd*]pyrene	2.8	7	3.0	7	77.7	5	6.3	8	7.3	3	69.5	6
Dibenzo[*ah*]anthracene	0.4	6	0.4	7	80.0	4	0.8	11	0.9	5	65.5	5
Dibenzo[*a,l*]pyrene	0.02	6	0.03	45	NC	NC	0.1	16	0.1	16	NC	NC
Dibenzo[*a,e*]pyrene	0.2	16	0.2	40	81.7	10	0.4	22	0.7	2	70.0	6
Dibenzo[*a,i*]pyrene	0.1	15	0.2	77	86.0	13	–^c^	–^c^	0.5	6	75.0	6
Dienzo[*a,h*]pyrene	0.1	14	0.2	67	NC	NC	–^c^	–^c^	0.4	9	NC	NC

Data are not blank-subtracted, ^a^ n = 5 replicates, ^b^ n = 3 replicates. ^c^ Only ^13^C standards were used

*IS* internal standard, *NC* not calculated—no internal standard for the indicated PAH), *NR* not reported

## Conclusions

In this study, three GC–MS systems were assessed for quantitative measurement of the 16 PAHs required by FDA (naphthalene, benzo[*c*]phenanthrene, benzo[*a*]anthracene, chrysene, cyclopenta-[*c,d*]pyrene, 5-methylchrysene, benzo[*b*]fluoranthene, benzo[*k*]fluoranthene, benzo[*j*]aceanthrylene, benzo[*a*]pyrene, indeno[*1,2,3*-*cd*]pyrene, dibenzo[*ah*]anthracene, dibenzo[*a,l*]pyrene, dibenzo[*a,e*]pyrene, dibenzo[*a,i*]pyrene, dibenzo[*a,h*]pyrene) in mainstream cigarette smoke.

Sample preparation strategy was improved by using exhaustive ASE extraction and a mixture of ethanol and toluene. The two-phase SPE clean up resulted in efficient removal of matrix artefacts. This allowed quantification of PAHs at very low levels using GC–HRMS, and probably also compensated for increased potential interference when low-resolution mass selective detection was used.

The GC separation conditions were the same for all three modes of detection and all three systems were equipped with a DB-EUPAH column, which is the optimal stationary phase for this separation. GC–HRMS detection system was found have the highest selectivity and sensitivity, providing a reduction in the interference of matrix co-extracts while achieving the lowest LOQs as compared with GC–MS/MS and GC–MS. Owing to the HR data acquisition mode enabling measurement of accurate mass, LOQs for PAHs were 5 to 15-fold lower for GC–HRMS than for GC–MS/MS and GC–MS.

These data demonstrate that the optimised sample preparation strategy followed by GC–HRMS analysis provides a fit-for-purpose and robust analytical approach, allowing fully quantitative determination of 16 PAHs and due to its robustness has a scope for further extension (both analytes and matrices/products), if required. Generation of such data is especially helpful where the toxicological assessment of the consumer exposure is missing or limited (all PAHs except naphthalene and B[*a*]P).

## Additional file

**Additional file 1.** Details of the chemical standards used for the analysis; MS acquisition parameters for all three GC/MS systems, PAH levels in blank samples together with their respective Limits of Detection (LODs) and Limits of Quantification (LOQs) as well as repeatability and accuracy of fortified Quality Control (QC) samples.

## Abbreviations

ASE: accelerated solvent extraction
B[*a*]P: benzo[a]pyrene
CFP: Cambridge filter pad
CO: carbon monoxide
CTP: Center for Tobacco Products
FDA: Food and Drug Administration
GC: gas chromatography
GC–HRMS: gas chromatography–high resolution mass spectrometry
GC–MS/MS: gas chromatography–tandem mass spectrometry
GC–MS: gas chromatography–mass spectrometry
HCI: Health Canada Intense
HPHC: harmful or potentially harmful constituent
HPLC: high-performance liquid chromatography
HPLC–MS/MS: high performance liquid chromatography–tandem mass spectrometry
HR: high resolution
IS: internal standard
LOD: limit of detection
LOQ: limit of quantification
MA: Massachusetts
MCS: mainstream cigarette smoke
MS: mass spectrometry
NA: not applicable
NFPDM: nicotine-free dry particulate matter (“tar”)
NR: not reported
PAHs: polycyclic aromatic hydrocarbons
QQQ: triple quadrupole
RMS: root-mean-square
RSD: relative standard deviation
S/N: signal to noise
SPE: solid-phase extraction
TPM: total particulate matter
UK: United Kingdom
US EPA: United States Environmental Protection Agency

## Footnotes

1 These were based on a United Kingdom Accreditation Service (UKAS)-accredited method (ISO 17025) for the analysis of PAH compounds by GC–HRMS (Marchwood Scientific Services, Southampton, UK).
