# Supplementary material for: Optimized method for determination of 16 FDA polycyclic aromatic hydrocarbons (PAHs) in mainstream cigarette smoke by gas chromatography–mass spectrometry
Source: Chem Cent J. 2018 Mar 13;12:27. doi: 10.1186/s13065-018-0397-2 (PMC5849724; doi:10.1186/s13065-018-0397-2)
Supplement: Supplementary file 1 — Additional file 1. Details of the chemical standards used for the analysis; MS acquisition parameters for all three GC/MS systems, PAH levels in blank samples together with their respective Limits of Detection (LODs) and Limits of Quantification (LOQs) as well as repeatability and accuracy of fortified Quality Control (QC) samples. [file 13065_2018_397_MOESM1_ESM.docx]

**Additional file**

**Optimized method for determination of 16 FDA polycyclic aromatic hydrocarbons (PAHs) in mainstream cigarette smoke by gas chromatography mass spectrometry**

Jana Jeffery^1^, Maria Carradus^2^, Karolina Songin^2^, Michael Pettit^2^, Karl Pettit^2^ and Christopher Wright^1^

^1^British American Tobacco, Research and Development, Southampton UK

^2^Marchwood Scientific Services, 371 Millbrook Rd W, Southampton, UK

This additional file includes details of the chemical standards used for the analysis; MS acquisition parameters for all three GC/MS systems, PAH levels in blank samples together with their respective Limits of Detection (LODs) and Limits of Quantification (LOQs) as well as repeatability and accuracy of fortified Quality Control (QC) samples.

**
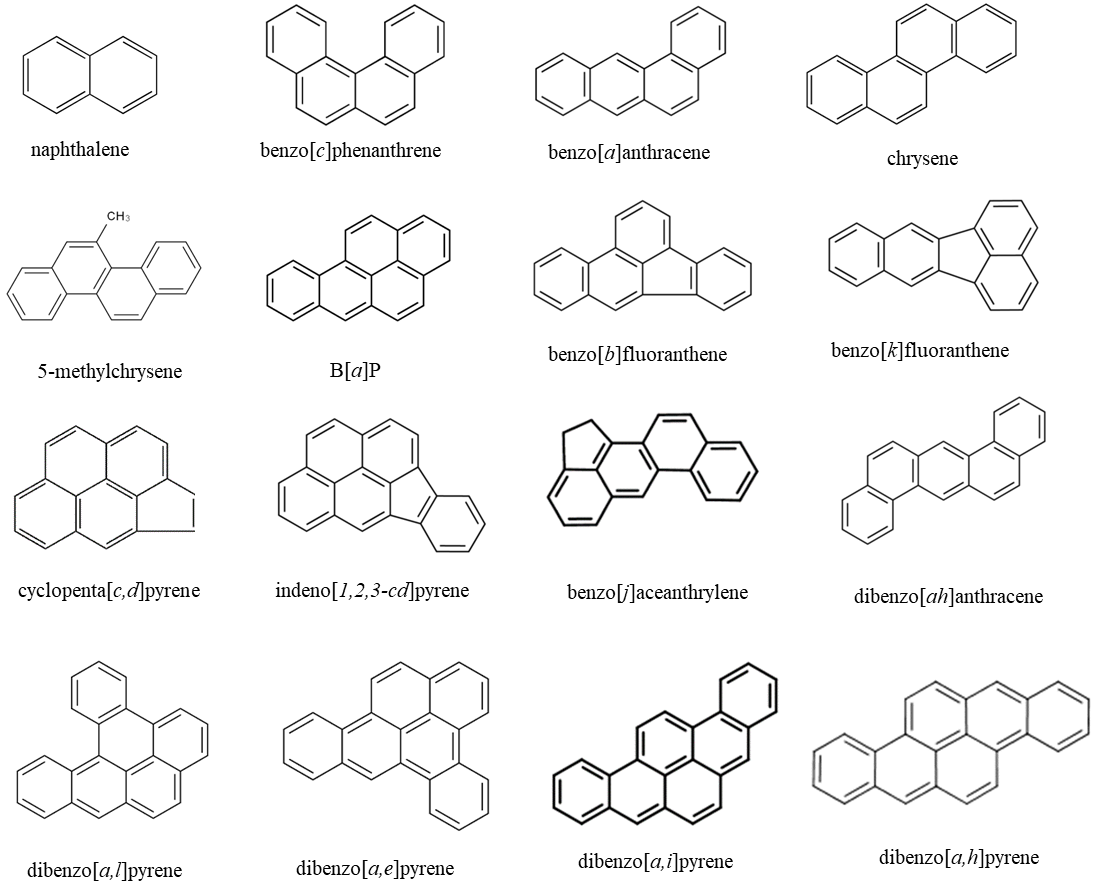
**

**Figure S1.** Structures of the 16 PAHs on FDA list of HPHCs

**Table S1.** Native and deuterated PAH and internal standards used in this study ^a^

| **Native PAHs** | **CAS#** | **Deuterated PAHs** | **CAS#** |
| --- | --- | --- | --- |
| Naphthalene | 91-20-3 | Naphthalene-d_8_ | 1146-65-2 |
| Acenaphthylene | 208-96-8 | Acenaphthene-d_10_ | 15067-26-2 |
| Acenaphthene | 83-32-9 |  |  |
| Fluorine | 86-73-7 | Fluorene-d_10_ | 81103-79-9 |
| Phenanthrene | 85-01-8 | Phenanthrene-d_10_ | 1517-22-2 |
| Anthracene | 120-12-7 | Anthracene-d_10_ | 1719-06-8 |
| Fluoranthene | 206-44-0 | Fluoranthene-d_10_ | 93951-69-0 |
| Pyrene | 129-00-0 | Pyrene-d_10_ | 1718-52-1 |
| Benz[*a*]anthracene | 56-55-3 | Benz[*a*]anthracene-d_12_ | 1718-53-2 |
| Cyclopenta[*c,d*]pyrene | 27208-37-3 |  |  |
| Chrysene | 218-01-9 | Chrysene-d_12_ | 1719-03-5 |
| 5-Methylchrysene | 3697-24-3 |  |  |
| Benzo[*b*]fluoranthene | 205-99-2 | Benzo[*b*]fluoranthene-d_12_ | 93951-98-5 |
| Benzo[*k*]fluoranthene | 207-08-9 | Benzo[*k*]fluoranthene-d_12_ | 93952-01-3 |
| Benzo[*j*]fluoranthene | 205-82-3 |  |  |
| Benzo[*a*]pyrene | 50-32-8 | Benzo[*a*]pyrene-d_12_ | 63466-71-7 |
| Indeno[*1,2,3-c,d*]pyrene | 193-39-5 | Indeno[*1,2,3-c,d*]pyrene-d_12_ | 203578-33-0 |
| Dibenz[*a,h*]anthracene | 53-70-3 | Dibenz[*a,h*]anthracene-d_14_ | 13250-98-1 |
| Benzo[*g,h,i*]perylene | 191-24-2 | Benzo[*g,h,i*]perylene-d_14_ | 93951-66-7 |
| Dibenzo[*a,l*]pyrene | 191-30-0 | Dibenzo[*a,l*]pyrene-d_14_ | 158776-07-9 |
| Dibenzo[*a,e*]pyrene | 192-65-4 |  |  |
| Dibenzo[*a,l*]pyrene | 189-55-9 |  |  |
| Dibenzo[*a,h*]pyrene | 189-64-0 |  |  |
|  |  | Deuterated internal standard |  |
|  |  | Acenaphthylene-d_8_ | 93951-97-4 |
|  |  | *p*-Terphenyl-d_14_ | 1718-51-0 |
|  |  | Benzo[*e*]pyrene-d_12_ | 205440-82-0 |
| ^a^ Product codes PAH-A-CS1, PAH-A-CS2, PAH-A-CS3, PAH-A-CS4 and PAH-A-CS5 (Wellington Laboratories, Guelph, Canada). | | | |

**Table S2.** ^13^C-labelled PAH standard mixture ^a^

| **Compound** | **Purity (%)** |
| --- | --- |
| Acenaphthene-^13^C_6_ | 100.0 |
| Acenaphthylene-^13^C_6_ | 98.0 |
| Anthracene-^13^C_6_ | 99.9 |
| Benz[*a*]anthracene-^13^C_6_ | 99.5 |
| Benzo[*b*]fluoranthene-^13^C_6_ | 99.8 |
| Benzo[*k*]fluoranthene-^13^C_6_ | 99.8 |
| Benzo[*g,h,i*]perylene-^13^C_12_ | 99.4 |
| Benzo[*a*]pyrene-^13^C_4_ | 98.9 |
| Chrysene-^13^C_6_ | 97.8 |
| Dibenz[*a,h*]anthracene-^13^C_6_ | 99.3 |
| Fluoranthene-^13^C_6_ | 99.9 |
| Fluorene-^13^C_6_ | 99.8 |
| Indeno[*1,2,3-c,d*]pyrene-^13^C_6_ | 97.7 |
| Naphthalene-^13^C_6_ | 99.0 |
| Phenanthrene-^13^C_6_ | 98.9 |
| Pyrene-^13^C_3_ | 98.6 |
| ^a^ Product code PAH-CVS-A (Wellington Laboratories, Guelph, Canada). | |

**Table S3.** GC–MS data acquisition parameters ^a^

| **Group (min)** | **Mass (*m/z*), dwell time (ms)** | | | | | |
| --- | --- | --- | --- | --- | --- | --- |
| 1, 0 | 89.00, 100 | 102.00, 100 | 118.00, 100 | 128.00, 100 | 133.00, 100 | 134.00, 100 |
| 2, 25.00 | 202.00, 100 | 224.00, 100 | 226.00, 100 | 228.00, 100 | 232.00, 100 | 234.00, 100 |
| 3, 30.50 | 224.00, 100 | 226.00, 100 | – | – | – | – |
| 4, 33.50 | 250.00, 100 | 252.00, 100 | 254.00, 100 | 256.00, 100 | 258.00, 100 |  |
| 5, 36.30 | 250.00, 100 | 252.00, 100 | 254.00, 100 | 256.00, 100 | – | – |
| 6, 45.00 | 274.00, 100 | 276.00, 100 | 278.00, 100 | 280.00, 100 | 282.00, 100 | 284.00, 100 |
| 7, 60.00 | 300.00, 100 | 302.00, 100 | 306.00, 100 | 308.00, 100 | 312.00, 100 | 314.00, 100 |

^a^ Selected ion monitoring (SIM) mode.

**Table S4.** GC–MS/MS data acquisition parameters

| **MS method** | **Precursor ion**  **(*m/z*)** | **Product ion 1**  **(*m/z*)** |
| --- | --- | --- |
| 1st time segment, t=0 min |  |  |
| Naphthalene | 128 | 102 |
| Naphthalene | 128 | 78 |
| ^13^C_6_-Naphthalene | 134.1 | 133 |
| 2nd time segment, t=25.00 min |  |  |
| Benzo[*c*]phenanthrene | 228 | 227 |
| Benzo[*c*]phenanthrene | – | 226 |
| Benzo[*a*]anthracene | 228.1 | 226.1 |
| Benzo[*a*]anthracene | 228.1 | 202.1 |
| ^13^C_6_-Benzo[*a*]anthracene | 234.1 | 232.1 |
| Chrysene | 228.1 | 226.1 |
| Chrysene | 228.1 | 202.1 |
| ^13^C_6_-Chrysene | 234.1 | 232.1 |
| Cyclopenta [*c,d*]pyrene | 226 | 224.1 |
| Cyclopenta [*c,d*]pyrene | 226 | 200.1 |
| 3rd time segment, t=30.50 min |  |  |
| 5-Methylchrysene | 242.1 | 239.1 |
| 5-Methylchrysene | 242.1 | 226.1 |
| 4th time segment, t=33.00 min |  |  |
| Benzo[*b*]fluoranthene | 252.1 | 250.1 |
| Benzo[*b*]fluoranthene | 252.1 | 226.1 |
| ^13^C_6_-Benzo[*b*]fluoranthene | 258.1 | 256.1 |
| Benzo[*k*]fluoranthene | 252.1 | 250.1 |
| Benzo[*k*]fluoranthene | 252.1 | 226.1 |
| ^13^C_6_-Benzo[*k*]fluoranthene | 258.1 | 256.1 |
| Benzo[*j*]aceanthrylene | 252 | 250 |
| Benzo[*j*]aceanthrylene | 252 | 226 |
| Benzo[*j*]aceanthrylene-C13 | 256 | 254 |
| 5th time segment, t=35.40 min |  |  |
| Benzo[*a*]pyrene | 252.1 | 250.1 |
| Benzo[*a*]pyrene | 252.1 | 226.1 |
| ^13^C_4_-Benzo[*a*]pyrene | 256.1 | 254.1 |
| 9,10,11,12-^13^C_4_-Benzo[*e*]pyrene | 256.1 | 254.1 |
| 6th time segment, t=45.00 min |  |  |
| Indeno[*1,2,3-c,d*]pyrene | 276.1 | 274.1 |
| Indeno[*1,2,3-c,d*]pyrene | 276.1 | 275.1 |
| Indeno[*1,2,3-c,d*]pyrene | 276.1 | 272.1 |
| ^13^C_6_-Indeno[*1,2,3-c,d*]pyrene | 282.1 | 280.1 |
| Dibenzo[*a,h*]anthracene | 278.1 | 276.1 |
| Dibenzo[*a,h*]anthracene | 278.1 | 252 |
| ^13^C_6_-Dibenzo[*ah*]anthracene | 284.2 | 282.1 |
| 7th time segment, t=60.00 min |  |  |
| Dibenzo[*a,l*]pyrene | 302.1 | 300.1 |
| Dibenzo[*a,l*]pyrene | 302.1 | 298.1 |
| Dibenzo[*a,e*]pyrene | 302.1 | 300.1 |
| Dibenzo[*a,e*]pyrene | 302.1 | 298.1 |
| ^13^C_6_-dibenzo[*a,e*]pyrene | 308.2 | 306.2 |
| Benzo[*a,i*]pyrene | 302.1 | 300.1 |
| Benzo[*a,i*]pyrene | 302.1 | 298.1 |
| ^13^C_12_-Dibenzo[*a,i*]pyrene | 314.2 | 312.2 |
| Dibenzo[*a,h*]pyrene | 302.1 | 300.1 |
| Dibenzo[*a,h*]pyrene | 302.1 | 298.1 |

**Table S5.** GC–MS/MS data quantification parameters

| **Data analysis method** | | |
| --- | --- | --- |
| **Native standard** | **Internal standard** | **Recovery standard** |
| Naphthalene | ^13^C_6_-Naphthalene | ^9,10,11,12-13^C_4_-Benzo[*e*]pyrene |
| Benzo[*c*]phenanthrene | ^13^C_6_-Benzo[*a*]anthracene | ^9,10,11,12-13^C_4_-Benzo[*e*]pyrene |
| Benzo[*a*]anthracene | ^13^C_6_-Benzo[*a*]anthracene | ^9,10,11,12-13^C_4_-Benzo[*e*]pyrene |
| Chrysene | ^13^C_6_-Chrysene | 9,10,11,12-^13^C_4_-Benzo[*e*]pyrene |
| Cyclopenta [*c,d*]pyrene | ^13^C_6_-Chrysene | 9,10,11,12-^13^C_4_-Benzo[*e*]pyrene |
| 5-Methylchrysene | ^13^C_6_-Chrysene | 9,10,11,12-^13^C_4_-Benzo[*e*]pyrene |
| Benzo[*b*]fluoranthene | ^13^C_6_-Benzo[*b*]fluoranthene | 9,10,11,12-^13^C_4_-Benzo[*e*]pyrene |
| Benzo[*k*]fluoranthene | ^13^C_6_-Benzo[*k*]fluoranthene | 9,10,11,12-^13^C_4_-Benzo[*e*]pyrene |
| Benzo[*j*]aceanthrylene | ^13^C_2_,d_2_-Benzo[*j*]aceanthrylene | 9,10,11,12-^13^C_4_-Benzo[*e*]pyrene |
| Benzo[*a*]pyrene | ^13^C_4_-Benzo[*a*]pyrene | 9,10,11,12-^13^C_4_-Benzo[*e*]pyrene |
| Indeno[*1,2,3-c,d*]pyrene | ^13^C_6_-Indeno[*1,2,3-c,d*]pyrene | 9,10,11,12-^13^C_4_-Benzo[*e*]pyrene |
| Dibenzo[*a,h*]anthracene | ^13^C_6_-Dibenzo[*a,h*]anthracene | 9,10,11,12-^13^C_4_-Benzo[*e*]pyrene |
| Dibenzo[*a,l*]pyrene | ^13^C_6_-dibenzo[*a,e*]pyrene | 9,10,11,12-^13^C_4_-Benzo[*e*]pyrene |
| Dibenzo[*a,e*]pyrene | ^13^C_6_-dibenzo[*a,e*]pyrene | 9,10,11,12-^13^C_4_-Benzo[*e*]pyrene |
| Benzo[*a,i*]pyrene | ^13^C_12_-Dibenzo[*a,i*]pyrene | 9,10,11,12-^13^C_4_-Benzo[*e*]pyrene |
| Dibenzo[*a,h*]pyrene | ^13^C_12_-Dibenzo[*a,i*]pyrene | 9,10,11,12-^13^C_4_-Benzo[*e*]pyrene |

**Table S6.** GC–HRMS data acquisition parameters

| **MS method** | **Ion** |
| --- | --- |
| 1st time segment, t=0 |  |
| Naphthalene | 128.0626 |
| d_8_-Naphthalene | 136.1128 |
| d_10_-Acenaphthene | 164.141 |
| 2nd time segment, t=25.00 |  |
| Benzo[*c*]phenanthrene | 228.0939 |
| Benzo[*a*]anthracene | 228.0939 |
| d_12_-Benzo[*a*]anthracene | 240.1692 |
| Chrysene | 228.0939 |
| d_12_-Chrysene | 240.1692 |
| Cyclopenta [*c,d*]pyrene | 226.0783 |
| 5-Methylchrysene | 242.1096 |
| 3rd time segment, t=30.50 |  |
| Benzo[*b*]fluoranthene | 252.0939 |
| d_12_-Benzo[*b*]fluoranthene | 264.1692 |
| Benzo[*k*]fluoranthene | 252.0939 |
| d_12_-Benzo[*k*]fluoranthene | 264.1692 |
| Benzo[*j*]aceanthrylene | 252.0939 |
| d_12_-Benzo[*e*]pyrene | 264.1692 |
| d_12_-Benzo[*a*]pyrene | 264.1692 |
| Benzo[*a*]pyrene | 252.0939 |
| 4th time segment, t=45.00 |  |
| Indeno[*1,2,3-c,d*]pyrene | 276.0939 |
| d_12_-Indeno[*1,2,3-c,d*]pyrene | 288.1692 |
| Dibenzo[*a,h*]anthracene | 278.1096 |
| d_14_-Dibenzo[*a,h*]anthracene | 292.1974 |
| 5th time segment, t=60.00 |  |
| Dibenzo[*a,l*]pyrene | 302.1096 |
| Dibenzo[*a,e*]pyrene | 302.1096 |
| Benzo[*a,i*]pyrene | 302.1096 |
| Dibenzo[*a,h*]pyrene | 302.1096 |
| **^13^Carbon** |  |
| Naphthalene | 128.0626 |
| ^13^C_6_-Naphthalene | 134.0827 |
| Benzo[*c*]phenanthrene | 228.0939 |
| Benzo[*a*]anthracene | 228.0939 |
| ^13^C_6_-Benzo[*a*]anthracene | 184.0984 |
| Chrysene | 228.0939 |
| ^13^C_6_-Chrysene | 234.114 |
| Cyclopenta [*c,d*]pyrene | 226.0783 |
| 5-Methylchrysene | 242.1096 |
| Benzo[*b*]fluoranthene | 252.0939 |
| ^13^C_6_-Benzo[*b*]fluoranthene | 258.114 |
| Benzo[*k*]fluoranthene | 252.0939 |
| ^13^C_6_-Benzo[*k*]fluoranthene | 258.114 |
| Benzo[*j*]aceanthrylene | 252.0939 |
| ^13^C2,d_2_-Benzo[*j*]aceanthrylene | 256.1073 |
| Benzo[*a*]pyrene | 252.0939 |
| ^13^C_4_-Benzo[*a*]pyrene | 256.1073 |
| 9,10,11,12-^13^C_4_-Benzo[*e*]pyrene | 256.1073 |
| Indeno[*1,2,3-c,d*]pyrene | 276.0939 |
| ^13^C_6_-Indeno[*1,2,3-c,d*]pyrene | 282.114 |
| Dibenzo[*a,h*]anthracene | 278.1096 |
| ^13^C_6_-Dibenzo[*a,h*]anthracene | 284.1297 |
| Dibenzo[*a,l*]pyrene | 302.1096 |
| Dibenzo[*a,e*]pyrene | 302.1096 |
| ^13^C_6_-dibenzo[*a,e*]pyrene | 314.1498 |
| Benzo[*a,i*]pyrene | 302.1096 |
| ^13^C_12_-Dibenzo[*a,i*]pyrene | 308.1297 |

**Table S7.** GC–HRMS data quantification parameters

| **Native standard** | **Deuterated standard** | **Internal standard** |
| --- | --- | --- |
| **Deuterated** |  |  |
| Naphthalene | d_8_-Naphthalene | d_10_-Acenaphthene |
| Benzo[*c*]phenanthrene | d_12_-Benzo[*a*]anthracene | d_10_-Acenaphthene |
| Benzo[*a*]anthracene | d_12_-Benzo[*a*]anthracene | d_10_-Acenaphthene |
| Chrysene | d_12_-Chrysene | d_10_-Acenaphthene |
| Cyclopenta [*c,d*]pyrene | d_12_-Chrysene | d_10_-Acenaphthene |
| 5-Methylchrysene | d_12_-Chrysene | d_10_-Acenaphthene |
| Benzo[*b*]fluoranthene | d_12_-Benzo[*b*]fluoranthene | d_12_-Benzo[*e*]pyrene |
| Benzo[*k*]fluoranthene | d_12_-Benzo[*k*]fluoranthene | d_12_-Benzo[*e*]pyrene |
| Benzo[*j*]aceanthrylene | d_12_-Benzo[*k*]fluoranthene | d_12_-Benzo[*e*]pyrene |
| Benzo[*a*]pyrene | d_12_-Benzo[*a*]pyrene | d_12_-Benzo[*e*]pyrene |
| Indeno[*1,2,3-c,d*]pyrene | d_12_-Indeno[1,2,3-cd]pyrene | d_12_-Benzo[*e*]pyrene |
| Dibenzo[*a,h*]anthracene | d_14_-Dibenzo[ah]anthracene | d_12_-Benzo[*e*]pyrene |
| Dibenzo[*a,l*]pyrene | d_14_-Dibenzo[ah]anthracene | d_12_-Benzo[*e*]pyrene |
| Dibenzo[*a,e*]pyrene | d_14_-Dibenzo[ah]anthracene | d_12_-Benzo[*e*]pyrene |
| Benzo[*a,i*]pyrene | d_14_-Dibenzo[ah]anthracene | d_12_-Benzo[*e*]pyrene |
| Dibenzo[*a,h*]pyrene | d_14_-Dibenzo[ah]anthracene | d_12_-Benzo[*e*]pyrene |
| **^13^Carbon** |  |  |
| Naphthalene | ^13^C_6_-Naphthalene | ^9,10,11,12-13^C_4_-Benzo[*e*]pyrene |
| Benzo[*c*]phenanthrene | ^13^C_6_-Benzo[*a*]anthracene | ^9,10,11,12-13^C_4_-Benzo[*e*]pyrene |
| Benzo[*a*]anthracene | ^13^C_6_-Benzo[*a*]anthracene | ^9,10,11,12-13^C_4_-Benzo[*e*]pyrene |
| Chrysene | ^13^C_6_-Chrysene | ^9,10,11,12-13^C_4_-Benzo[*e*]pyrene |
| Cyclopenta [*c,d*]pyrene | ^13^C_6_-Chrysene | 9,10,11,12-^13^C_4_-Benzo[*e*]pyrene |
| 5-Methylchrysene | ^13^C_6_-Chrysene | 9,10,11,12-^13^C_4_-Benzo[*e*]pyrene |
| Benzo[*b*]fluoranthene | ^13^C_6_-Benzo[*b*]fluoranthene | 9,10,11,12-^13^C_4_-Benzo[*e*]pyrene |
| Benzo[*k*]fluoranthene | ^13^C_6_-Benzo[*k*]fluoranthene | 9,10,11,12-^13^C_4_-Benzo[*e*]pyrene |
| Benzo[*j*]aceanthrylene | ^13^C2,d2-Benzo[*j*]aceanthrylene | 9,10,11,12-^13^C_4_-Benzo[*e*]pyrene |
| Benzo[*a*]pyrene | ^13^C_4_-Benzo[*a*]pyrene | 9,10,11,12-^13^C_4_-Benzo[*e*]pyrene |
| Indeno[1,2,3-cd]pyrene | ^13^C_6_-Indeno[1,2,3-cd]pyrene | 9,10,11,12-^13^C_4_-Benzo[*e*]pyrene |
| Dibenzo[ah]anthracene | ^13^C_6_-Dibenzo[ah]anthracene | 9,10,11,12-^13^C_4_-Benzo[*e*]pyrene |
| Dibenzo[*a,l*]pyrene | ^13^C_6_-dibenzo[*a,e*]pyrene | 9,10,11,12-^13^C_4_-Benzo[*e*]pyrene |
| Dibenzo[*a,e*]pyrene | ^13^C_6_-dibenzo[*a,e*]pyrene | 9,10,11,12-^13^C_4_-Benzo[*e*]pyrene |
| Benzo[*a,i*]pyrene | ^13^C_12_-Dibenzo[*a,i*]pyrene | 9,10,11,12-^13^C_4_-Benzo[*e*]pyrene |
| Dibenzo[*a,h*]pyrene | ^13^C_12_-Dibenzo[*a,i*]pyrene | 9,10,11,12-^13^C_4_-Benzo[*e*]pyrene |

**Table S8.** Quality controls analysed by GC-HRMS ^a^

| **PAH** | **QC (n=3)** | | |
| --- | --- | --- | --- |
|  | **Average**  **(ng/CFP)** | **RSD**  **(%)** | **Recovery**  **(%)** |
| Naphthalene | 44.8 | 5.18 | 112 |
| Benzo[*c*]phenanthrene | 41.2 | 1.65 | 103 |
| Benzo[*a*]anthracene | 39.3 | 1.26 | 98.2 |
| Chrysene | 40.3 | 3.69 | 101 |
| Cyclopenta-[*c,d*]pyrene | 41.6 | 2.19 | 104 |
| 5-methylchrysene | 43.8 | 3.57 | 110 |
| Benzo[*b*]fluoranthene | 40.8 | 2.11 | 102 |
| Benzo[*k*]fluoranthene | 40.0 | 2.56 | 99.9 |
| Benzo[*j*]aceanthrylene | 39.4 | 3.27 | 98.5 |
| Benzo[*a*]pyrene | 41.6 | 0.87 | 104 |
| Indeno[*1,2,3*-*cd*]pyrene | 40.0 | 0.99 | 100 |
| Dibenzo[*ah*]anthracene | 40.1 | 2.69 | 100 |
| Dibenzo[*a,l*]pyrene | 39.7 | 2.21 | 99.1 |
| Dibenzo[*a,e*]pyrene | 38.7 | 3.25 | 96.7 |
| Dibenzo[*a,i*]pyrene | 38.1 | 7.22 | 95.2 |
| Dibenzo[*a,h*]pyrene | 39.9 | 5.24 | 99.8 |
| CFPs fortified with 40 ng of native standards and 100 ng of internal standards. | | | |

**Table S9.** Mean apparent recovery of C13 internal standards in 3R4F MCS extracts

| PAH compound | ISO smoking regime | | | | | |  | | HCI smoking regime | | | | | |  | |
| --- | --- | --- | --- | --- | --- | --- | --- | --- | --- | --- | --- | --- | --- | --- | --- | --- |
|  | GC-HRMS | | GC-MS/MS | | | GC-MS | | | GCHRMS | | GC-MS/MS | | | GC-MS | | |
|  | Mean recovery (%) | SD | | Mean recovery (%) | SD | Mean recovery (%) | | SD | Mean recovery (%) | SD | | Mean recovery (%) | SD | Mean recovery (%) | | SD |
| 13C6-Naphthalene | 73 | 7.0 | | 8 | 0.2 | 133 | | 61.8 | 73 | 4 | | 14 | 0.3 | 183 | | 52.8 |
| 13C6-Benzo(a)anthracene | 81 | 3.0 | | 51 | 20.8 | 100 | | 8.8 | 87 | 10 | | 172 | 2.6 | 106 | | 4.2 |
| 13C6-Chrysene | 59 | 4.0 | | 82 | 1.4 | 100 | | 15.6 | 75 | 11 | | 145 | 3.1 | 99 | | 0.7 |
| 13C6-Benzo(b)fluoranthene | 79 | 4.0 | | 65 | 4.7 | 97 | | 6.0 | 92 | 12 | | 83 | 1.8 | 105 | | 2.7 |
| 13C6-Benzo(k)fluoranthene | 83 | 3.0 | | 67 | 6.0 | 105 | | 7.3 | 92 | 11 | | 84 | 2.0 | 109 | | 2.3 |
| 13C2-Benzo(j)aceanthrylene | 66 | 8.0 | | 14 | 1.1 | 38 | | 2.2 | 82 | 5 | | 17 | 0.4 | 40 | | 1.6 |
| 13C4-Benzo(a)pyrene | 75 | 3.0 | | 64 | 2.8 | 83 | | 9.5 | 85 | 6 | | 66 | 2.1 | 101 | | 8.2 |
| 13C6-Indeno(1,2,3-cd)pyrene | 78 | 5.0 | | 20 | 1.9 | 46 | | 2.1 | 70 | 6 | | 14 | 0.5 | 69 | | 3.2 |
| 13C6-Dibenzo(ah)anthracene | 80 | 4.0 | | 29 | 1.6 | 71 | | 27.2 | 66 | 5 | | 19 | 0.4 | 81 | | 2.2 |

**SD -Standard Deviation (number of replicates - 3)*

**Table S10.** Mean apparent recovery of deuterated internal standards in 3R4F MCS extracts

| PAH compound | ISO smoking regime | | | | | |  | | HCI smoking regime | | | | |  | |
| --- | --- | --- | --- | --- | --- | --- | --- | --- | --- | --- | --- | --- | --- | --- | --- |
|  | GC-HRMS | | | GC-MS/MS | | GC-MS | | | GCHRMS | | GC-MS/MS | | GC-MS | | |
|  | Mean recovery (%) | | SD | Mean recovery (%) | SD | Mean recovery (%) | | SD | Mean recovery (%) | SD | Mean recovery (%) | SD | Mean recovery (%) | | SD |
| D8-Naphthalene | | 68 | 6.8 | 22 | 0.5 | 64 | | 24.1 | 65 | 7.0 | 43 | 1.4 | 64 | | 4.8 |
| D12-Benzo(a)anthracene | | 73 | 12.0 | 32 | 0.2 | 99 | | 6.8 | 72 | 12.0 | 128 | 4.2 | 104 | | 2.1 |
| D12-Chrysene | | 71 | 5.8 | 78 | 2.4 | 105 | | 10.0 | 70 | 10.0 | 139 | 4.1 | 113 | | 3.5 |
| D12-Benzo(b)fluoranthene | | 62 | 12.6 | 59 | 3.5 | 89 | | 4.4 | 83 | 14.0 | 74 | 2.2 | 94 | | 1.8 |
| D12-Benzo(k)fluoranthene | | 75 | 12.5 | 62 | 4.8 | 95 | | 4.0 | 82 | 13.0 | 76 | 2.3 | 101 | | 3.8 |
| D12-Benzo(a)pyrene | | 69 | 9.3 | 64 | 3.0 | 80 | | 1.7 | 78 | 8.0 | 65 | 1.3 | 85 | | 5.9 |
| D14-Dibenzo(ah)anthracene | | 72 | 7.7 | 20 | 1.5 | 51 | | 1.6 | 63 | 6.0 | 13 | 0.3 | 57 | | 1.5 |
| D12-Indeno(1,2,3-cd)pyrene | | 74 | 5.5 | 19 | 1.5 | 65 | | 2.6 | 61 | 7.0 | 12 | 0.3 | 71 | | 1.7 |

**SD -Standard Deviation (number of replicates - 5)*
